# SARS-CoV-2 Spike-Heat Shock Protein A5 (GRP78) Recognition may be Related to the Immersed Human Coronaviruses

**DOI:** 10.3389/fphar.2020.577467

**Published:** 2020-12-11

**Authors:** Abdo A. Elfiky

**Affiliations:** Biophysics Department, Faculty of Science, Cairo University, Giza, Egypt

**Keywords:** GRP78, HSPA5, SARS-CoV-2, viral attachment, spike, host-cell recognition, COVID-19, cross-vaccination

## Abstract

The human coronavirus (HCoV), SARS-CoV-2, caused more than 34 M confirmed infections from which more than 1 M deaths are reported until now (the WHO situation report-154). The current pandemic causes severe socio-economic burden. Due to the importance of understanding of the mode of recognition and viral entry, spike protein shed drug designers as the first look protein target with the first released solved structure on 26 February 2020 (PDB ID: 6VSB). It is proposed that the recognition site for GRP78 is found in SARS-CoV-2 and the immersed human coronaviruses but experimental validation is still required.

The human coronavirus (HCoV), SARS-CoV-2, caused more than 34 M confirmed infections from which ≥ 1 M deaths are reported until now (the WHO situation report-154). The current pandemic causes severe socio-economic burden (Nicola et al., 2020). Due to the importance of understanding of the mode of recognition and viral entry, spike protein sheds drug designers as the first look protein target. The first solved structure for the spike protein is released on February 26, 2020 (PDB ID: 6VSB) (Wrapp et al., 2020).

Heat Shock Protein A5 (HSPA5), or the Glucose Regulating Protein 78 (GRP78), was reported to be a possible route for SARS-CoV-2 attachment and entry (Elfiky, 2020; Ibrahim et al., 2020; Ha et al., 2020; Saghazadeh and Rezaei, 2020). The binding site in the SARS-CoV-2 spike is predicted to be the nine residues CNGVEGFNC (C480-C488 region) found in the S1 C-terminal domain, as shown in Figure 1 (see the enlarged panel) (Ibrahim et al., 2020). The prediction was based on the structural conservation between this region of the spike, and the cyclic Pep42 peptide (CTVALPGGYVRVC) that previously reported to target GRP78 (over cancer cells) selectively *in vivo* (Kim et al., 2006; Quinones et al., 2008; Braun et al., 2020). It was recently reported in Nature journal that SARS-CoV-2 spike has conserved motifs compared to previous strains of human coronaviruses (HKU1, 229E, NL63, OC43) (Braun et al., 2020) still experimental validation is required to prove the hypothesis.

**FIGURE 1:**
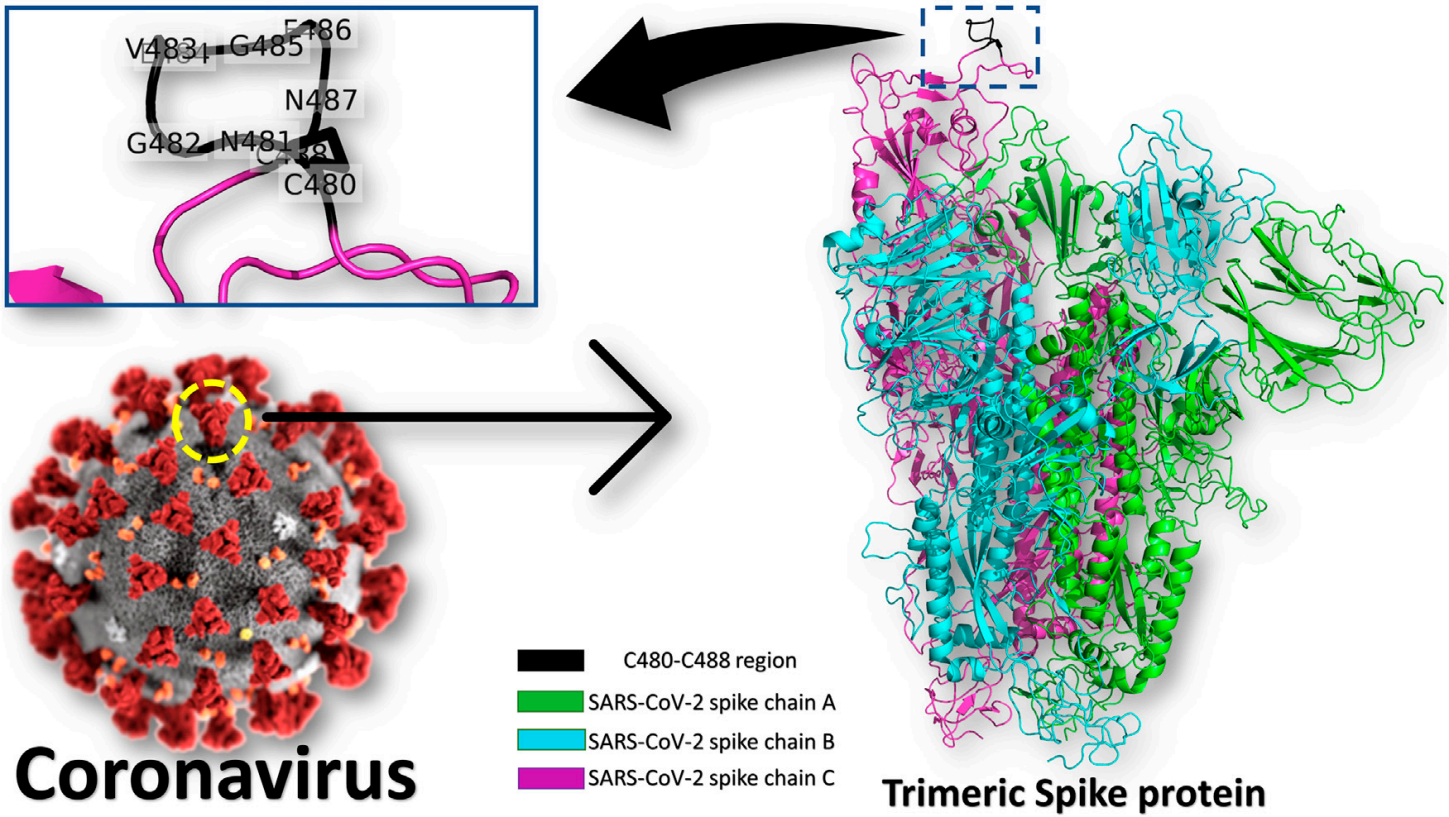
Coronavirus spike protein and GRP78 recognition site. The trimeric spike protein (green, cyan, and magenta cartoons) is shown in colored cartons, while the GRP78 recognition site (C480-C488 in SARS-CoV-2) is depicted in the black cartoon (see the enlarged panel). The GRP78 recognizing residues in SARS-CoV-2 are labeled in the enlarged panel.

The sequence comparison between the known human coronavirus strains, including; *alpha coronaviruses* (NL63, and 229E), and *beta coronaviruses* (OC43, HKU1, MERS-CoV, SARS-CoV, and SARS-CoV-2), reveals exciting results in terms of C480-C488 region of the spike protein. Figure 2A shows part of the multiple sequence alignment, made by clustal omega webserver and viewed by ESpript 3, for the different human coronavirus strains at the GRP78 recognition site (C480-C488 in SARS-CoV-2). The recognition of the GRP78 by the peptide Pep42 is previously reported to be restricted for the cyclic form of the peptide (the first and last CYS residues form a disulfide bond) (Kim et al., 2006; Quinones et al., 2008). Surprisingly, the residues C480 and C488 is found in SARS-CoV-2 and the immersed human coronaviruses strains (NL63, 229E, OC43, and HKU1), but missing in SARS-CoV and MERS-CoV. Additionally, at least three identical (four similar) residues are found among the immersed human coronaviruses strains compared to SARS-CoV-2 (see Figure 2B). This implies the possibility of cross immune the SARS-CoV-2 and the immersed human coronaviruses strains (NL63, 229E, OC43, and HKU1).

**FIGURE 2:**
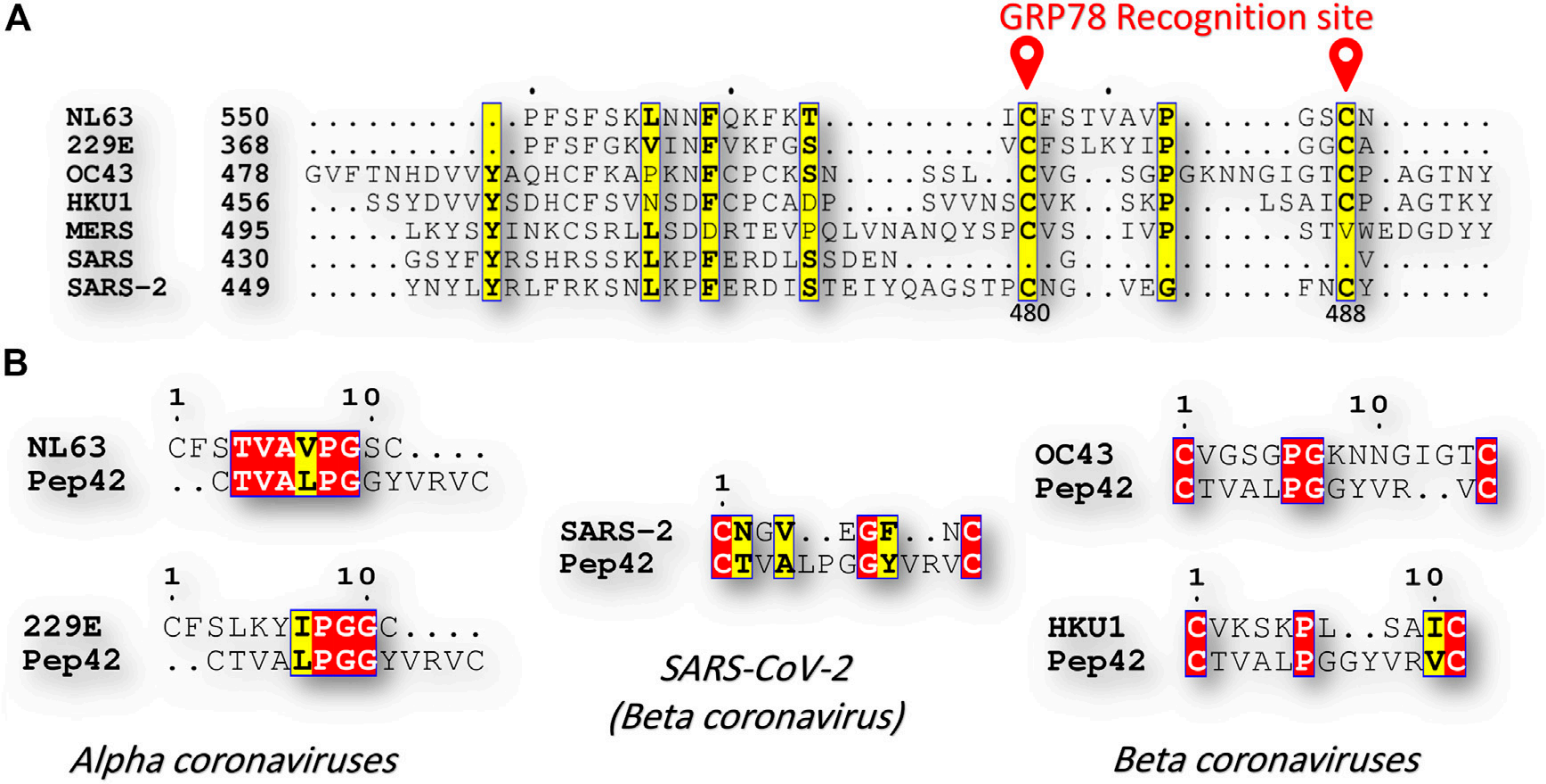
Sequence comparison **(A)** Part of the multiple sequence alignment for the spike protein of all of the currently reported human coronaviruses strains (NL63, 229E, OC43, HKU1, MERS-CoV, SARS-CoV, and SARS-CoV-2). The alignment is made using the Clustal Omega web server and is displayed by ESpript 3 software. The yellow highlighted residues are conserved among the seven HCoVs. GRP78 recognition site (C480-C488 in SARS-CoV-2) is marked in red **(B)** Pairwise sequence alignment between Pep42 from one side and NL63, 229E, OC43, HKU1, and SARS-CoV-2 from the other side. Red and yellow residues are identical and similar residues, respectively.

The human coronaviruses NL63, 229E, OC43, and HKU1, have been less impacted the human being (Zumla et al., 2016; van den Brand et al., 2015). People previously infected with these strains of human coronaviruses may develop immunity against SARS-CoV-2. Unfortunately, the immersed human coronavirus strains characterized by mild flu-like symptoms and information about viral distribution is rare (Huynh et al., 2012; Hilgenfeld and Peiris, 2013; Zumla et al., 2016).

Molecular dynamics study (using NAMD software) for the SARS-CoV-2 spike combined with molecular docking (Using AutoDock Vina software) revealed the existence of at least four interactions (H-bonds or hydrophobic contacts) between GRP78 and C480-C488 of SARS-CoV-2 spike. The hydrophobic contacts (two up to six) are found in all the docking experiments (seven replicas done at different dynamics states of SARS-CoV-2 spike after 100 ns MDS). This is in support of the previous reports about GRP78 recognition of hydrophobic patches in the unfolded proteins (Tsai and Lee, 2018; Li and Lee, 2006). Further experimental validation is required to prove this hypothesis.

Conclusively SARS-CoV-2 Spike-host cell recognition is crucial in fighting COVID-19 both as a therapeutic and prophylactic routes. The C480-C488 region is an essential viral spike epitope to be targeted by drugs, natural compounds, or antibodies to prevent or weaken the host cell recognition of SARS-CoV-2. This represents a hot topic that need further validation in laboratory.

## Data Availability Statement

The raw data supporting the conclusions of this article will be made available by the authors, without undue reservation.

## Author Contributions

AE own the research idea and wrote the manuscript.

## Conflict of Interest

The author declares that the research was conducted in the absence of any commercial or financial relationships that could be construed as a potential conflict of interest.
